# Comparison between a nurse-led weaning protocol and weaning based on physician’s clinical judgment in tracheostomized critically ill patients: a pilot randomized controlled clinical trial

**DOI:** 10.1186/s13613-018-0354-1

**Published:** 2018-01-22

**Authors:** Nazzareno Fagoni, Simone Piva, Elena Peli, Fabio Turla, Elisabetta Pecci, Livio Gualdoni, Bertilla Fiorese, Frank Rasulo, Nicola Latronico

**Affiliations:** 1 Department of Anesthesia, Critical Care and Emergency, Spedali Civili University Hospital, Piazzale Ospedali Civili, 1, 23123 Brescia, Italy; 2 Department of Molecular and Translational Medicine, University of Brescia, Brescia, Italy; 3 School of Specialty in Anesthesia, Intensive Care and Pain Medicine, University of Brescia, Brescia, Italy; 4 Department of Medical and Surgical Specialties, Radiological Sciences and Public Health, University of Brescia, Brescia, Italy

**Keywords:** Tracheostomy, Weaning, Mechanical ventilation, Nurse-led weaning protocol

## Abstract

**Background:**

Weaning protocols expedite extubation in mechanically ventilated patients, yet the literature investigating the application in tracheostomized patients remains scarce. The primary objective of this parallel randomized controlled pilot trial (RCT) was to assess the feasibility and safety of a nurse-led weaning protocol (protocol) compared to weaning based on physician’s clinical judgment (control) in tracheostomized critically ill patients.

**Results:**

We enrolled 65 patients, 27 were in the protocol group and 38 in the control group. Of 27 patients in the protocol group, 1 (3.7%) died in the ICU, 24 (88.9%) were successfully weaned from tracheostomy, and 2 (7.4%) were transferred still on the ventilator. Of 38 patients in the control group, 2 (5.3%) died in the ICU, 22 (57.9%) were successfully weaned from tracheostomy, and 14 were transferred still on the ventilator (36.8%). Risk of being discharged from the ICU on the ventilator was higher in the control group (relative risk: 1.5, IC 95% 1.14–2.01). Concerning safety and feasibility, no patients were excluded after randomization. There was no crossover between the two study arms nor missing data, and no severe adverse event related to the study protocol application was recorded by the staff. Weaning time and rate of successful weaning were not different in the protocol group compared to the control group (long-rank test, *p* = 0.31 for MV duration, *p* = 0.45 for weaning time). Based on our results and assuming a 30% reduction of the weaning time for the protocol group, 280 patients would be needed for a RCT to establish efficacy.

**Conclusions:**

In this pilot RCT we demonstrated that a nurse-led weaning protocol from tracheostomy was feasible and safe. A larger RCT is justified to assess efficacy.

**Electronic supplementary material:**

The online version of this article (10.1186/s13613-018-0354-1) contains supplementary material, which is available to authorized users.

## Background

Tracheostomy is a common practice in the intensive care unit (ICU), particularly in acutely ill neurological patients and those predicted to have long-term mechanical ventilation (MV) [1, 2]. Tracheostomy eliminates the risk of extubation failure and re-intubation, but may leave unaffected the process of withdrawing MV. Withdrawing MV can be difficult in these patients because those submitted to tracheostomy often have high-severity disease, complicated clinical course and predicted prolonged MV and ICU stay [1]. Therefore, improving weaning from tracheostomy can be a relevant clinical outcome. Weaning from MV is challenging and represents 40–50% of the duration of MV in patients with tracheal intubation and MV [3–5]. Weaning protocols [4, 6–8], automated systems [9] and daily spontaneous breathing trial (SBT) [10–13] have been proven to significantly reduce the duration of the weaning period and the total number of days on MV [4, 14–18]. However, very few efficacy studies have assessed therapeutic strategies to improve weaning from tracheostomy in the ICU. Vaschetto et al. [19] compared a systematic approach based on daily screening of meaningful physiologic and clinical variables followed by SBT with direct disconnection from the ventilator based on the sole physician’s judgment in reducing the rate of reconnection to the ventilator within 48 h; however, the trial was prematurely interrupted due to a high rate of reconnections. Jubran et al. [20] compared pressure support with unassisted breathing through an oxygen-delivery device in tracheostomized patients, but the trial was performed after patient discharge from the ICU to a long-term acute care hospital.

In our center, we strongly support the identification of strategies to improve ICU staff communication and coordination of care delivery as a key translational research priority for critical care [21, 22]. Weaning has been a top research priority in respiratory nursing for many years. Hence, the local weaning working group established that a nurse-led tracheostomy weaning protocol would be a clinically relevant, achievable goal. We therefore conducted the present pilot study to address whether an RCT concerning a nurse-led weaning protocol in tracheostomized patients represents an appropriate trial design, and whether it is feasible to decrease the weaning time and the duration of MV compared to weaning based on the physician’s clinical judgment alone. The feasibility objectives were: (1) ability of the ICU team to recruit a sufficient number of patients, (2) the rate of crossover between the two study arms, (3) the ability of the study team to collect data, (4) any severe adverse event during protocol application and (5) providing data to estimate the parameters required to design a definitive RCT. Other clinically relevant secondary outcomes evaluated were: the weaning time, duration of MV, successful weaning rate and the ICU length of stay (ICU-LOS).

## Methods

### Study design

This single-center parallel randomized controlled clinical pilot trial (ClinicalTrials.gov Identifier: NCT01877850) was conducted from May 2013 to May 2014 at the general and neurological ICU of the Department of Anesthesia, Critical Care and Emergency of the Spedali Civili of Brescia, a large regional university-affiliated hospital. The ICU has 10 beds, 6 general and 4 neurological. The daytime staffing of the unit consists of 1 medical coordinator, 1 attending physician, 2 residents (fourth- and fifth-year residents of the School of Specialty in Anesthesia and Critical Care Medicine) and 6 critical care nurses. The nightshift team consists of 1 attending physician, 1 resident and 4 nurses. Physical therapists are not dedicated exclusively to the ICU and provide general and respiratory physical therapy for 5 days a week based on a physiatrist-activated written protocol. We do not have respiratory therapists in the ICU.

Patients and those who performed the statistical analyses were blinded to the treatment assignment. The study was conducted in accordance with the Declaration of Helsinki. Ethical approval was obtained from the local ethics committee (registration number 1351/2013). Detailed written information was provided to the patients and family members about the study, and written informed consent to participate in the study was obtained from the patient whenever possible. In case of altered consciousness, the ethics committee waived the requirement for consent; according to Italian laws relatives are not regarded as legal representatives of the patient in the absence of a formal designation [23]. Written informed consent was subsequently requested from all surviving patients as soon as they regained their mental competency. The study conforms to CONSORT extension for pilot studies [24].

### Study participants

ICU patients were included if they were ≥ 18 years and had received a tracheostomy during their ICU stay. We excluded patients who were expected to die soon, for whom simple weaning was predicted, those already tracheostomized at ICU admission and those with pre-existing central and peripheral nervous system degenerative diseases with an increased risk of permanent home ventilation.

All patients on MV through an orotracheal tube and the patients in the control group were managed by the attending physician using standard care [25]. In particular (Additional file 1: eFigure 1), after spontaneous awakening trial (SAT), the attending physician performed an SBT screening and started the SBT trial. If SBT trial (using a T-piece) was passed, the patient was extubated; otherwise, the patient was reconnected to the ventilator with the previous setting. When the safety screen (Additional file 1: eFigure 1) was not met, the attending physician attempted to reduce the ventilator settings based on his/her clinical judgment.

Patients predicted to have: (1) prolonged MV (more than 21 days), (2) difficult weaning from the ventilator or (3) prolonged weaning (as defined by the Statement of the Sixth International Consensus Conference on Intensive Care Medicine [26]), received a tracheostomy. The medical ICU staff (medical coordinator and attending physician) was responsible for providing the indication for tracheostomy based on accurate clinical evaluation (admission diagnosis, severity, patients demographic characteristics) and in consideration of the current available literature [27–30].

### Study procedures

All tracheostomized patients enrolled into the study were randomly assigned either to the nurse-led weaning group (protocol group) or to the weaning group based on physician’s clinical judgment (control group). Randomization sequence was created using Excel 2013 (Microsoft, Redmond, WA, USA) with simple allocation [31] to the two study groups. The allocation sequence was concealed from the researcher enrolling and assessing participants in sequentially numbered, opaque, sealed and stapled envelopes and securely stored.

### Interventions

**Fig. 1:**
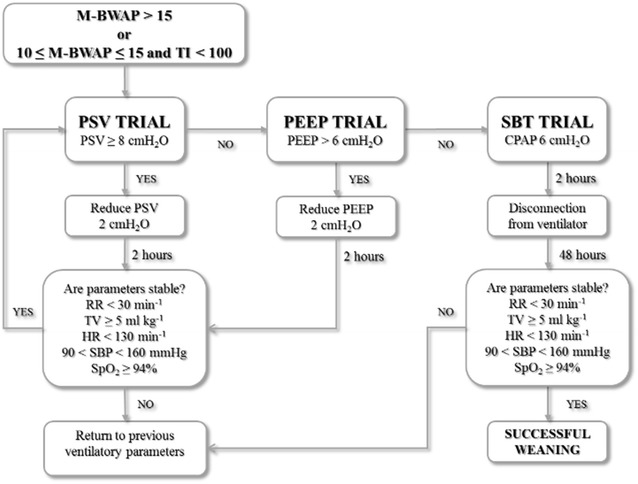
The nurse-led weaning protocol for tracheostomized critically ill patients. The figure shows the pressure support ventilation (PSV) trial, positive end-expiratory pressure (PEEP) trial and spontaneous breathing trial (SBT). *CPAP* continuous positive airway pressure, *HR* heart rate, *M-BWAP* modified Burns Wean Assessment Program, *SBP* systolic blood pressure, *SpO*_*2*_ pulse oximeter oxygen saturation, *TI* Tobin Index (respiratory rate/tidal volume)

Routine care was no different between the two groups. Nurse training was necessary before starting the clinical trial, consisting of four 2-h meetings with simulated cases and during routine care; the ICU staff was also provided with written instructions for proper weaning protocol procedure (Fig. 1).

In the protocol group, clinical stability was assessed using a modified version of the Burns Wean Assessment Program score (M-BWAP, see Additional file 1: note and eTable 1) [32, 33] and the respiratory system performance using the Tobin Index (TI, ratio between respiratory rate and tidal volume, in liters) [34]. In the M-BWAP we did not consider the negative inspiratory pressure ≤ 20 cmH_2_O, the positive expiratory pressure ≥ 30 cmH_2_O, the vital capacity and the PaCO_2_ of approximately 40 mmHg with minute ventilation < 10 L/min, which were impractical to measure systematically at bedside in the ICU [35, 36]. Patients randomized to the protocol group began ventilator weaning if a M-BWAP score was > 15, or if the M-BWAP was between 10 and 15 with a concurrently measured TI of < 100. Once the M-BWAP score was judged to be adequate, the nurses took charge of the weaning process as dictated by the protocol (Fig. 1). The protocol consisted of gradually decreasing *pressure support ventilation* (PSV) by 2 cmH_2_O, with a maximum of two steps per day (Fig. 1, PSV trial). When a patient tolerated a PSV < 8 cmH_2_O for at least 2 h, the nurse reduced the *positive end-expiratory pressure* (PEEP) by 2 cmH_2_O, with a maximum of two steps per day (Fig. 1, PEEP trial). Once the patient reached PSV < 8 cmH_2_O and PEEP < 8 cmH_2_O, the SBT took place, consisting of *continuous positive airways pressure* ventilation with 6 cmH_2_O for at least 2 h followed by disconnection from ventilator. At the end of the two steps, the respiratory rate, tidal volume, heart rate, blood pressure and peripheral oxygen saturation were monitored, and if one or more of these parameters were impaired, the ventilatory settings previously modified were restored.

Blood gas analysis was performed two times per day in both groups, in the morning, before starting any modifications of the MV, and in the evening.

### Feasibility outcome

Concerning the feasibility objectives, the primary outcome of the study, the ability of the ICU team to recruit the patients was evaluated by tracking all patients admitted to the ICU and reporting the number of patients screened by the study team and the percentage of the enrolled patients. Data concerning the rate of crossover between the study arms were also collected. The ability of the study team to collect data was assessed by measuring the missing value of the recorded variables at the end of the study. Any severe adverse event judged as being directly correlated to the protocol application—any major cardiac events (cardiac arrest, hemodynamically significant arrhythmias, acute coronary syndrome), or respiratory event (respiratory arrest)—was eventually recorded.

### Secondary outcomes (clinical outcomes)

Secondary clinical outcomes were the weaning time and the total duration of mechanical ventilation. Weaning time was defined as the number of days between the start of the weaning process and patient’s disconnection from the ventilator. In the protocol group, the weaning process started when the M-BWAP was > 15 or the M-BWAP was between 10 and 15 with the TI < 100; in the control group, the weaning process started when the on-call physician reduced PSV or PEEP on the ventilator for the first time with a declared intention to start weaning.

Weaning was considered successful when patients could breathe without ventilator assistance for at least 48 h; otherwise, the weaning was considered failed. In this case, the subject remained in the same study arm to which he was initially allocated; as soon as the clinical condition improved, a new weaning process took place. In case of weaning failure, the weaning time was computed until withdrawing from MV.

Duration of MV was defined as the difference between the beginning and the end of MV. The beginning of MV corresponded to the first day of orotracheal intubation. If a patient died or was discharged while still ventilated, the duration of weaning and that of MV were calculated up to the last day in the ICU.

### Statistical analysis

The following variables were recorded in an e-CRF and compared between groups: gender, age, the cause of ICU admission and the Simplified Acute Physiology Score II score at ICU admission; PaO_2_/FiO_2_ ratio, PSV value, PEEP value and M-BWAP score at the study enrollment for both groups. In the protocol group, the M-BWAP was calculated for each weaning trial (in case of weaning failure). Data were analyzed using R (version 3.3.3). All data analysis was carried out according to a pre-established intention-to-treat analysis plan. Data are presented as median (interquartile range) for ordinal variables or non-normally distributed continuous variables or as mean (standard deviation) for the normally distributed continuous variables. Normality was assessed by Kolmogorov–Smirnov test. Dichotomous data were compared by two-tailed *χ*^2^ test with the Yates correction or Fisher’s exact test when appropriate. Continuous variables were compared using the Mann–Whitney *U* test or *t* test as appropriate. A two-sided significance test was set at *p* < 0.05.

As primary analysis, we performed a Kaplan–Meier with log-rank test for weaning time and duration of MV, censoring the patients discharged from the ICU still ventilated or dead in ICU. Since we did not record follow-up data after ICU discharge, and hence we could not define the weaning outcome of those patients discharged while still ventilated, we performed a secondary analysis considering the patients still ventilated at ICU discharge as weaned. Since patients still ventilated when discharged from the ICU were less common among the study group, this categorization favored controls. Therefore, we hypothesized that if a significant reduction in the weaning time could be observed in the study arm, this would not be a chance finding.

## Results

**Fig. 2:**
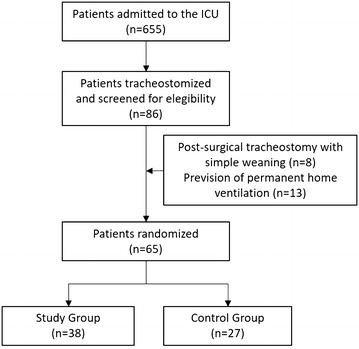
Patients flowchart. *ICU* intensive care unit. Post-surgery indicates patients receiving tracheostomy for surgical reasons (i.e., after maxillo-facial or head–neck surgery)

Of 655 patients admitted to the ICU, 86 (13.1%) received a percutaneous tracheostomy and were screened for eligibility. Of these, 65 (77%) met the inclusion criteria and were randomized to the protocol group (27) or to the control group (38) (Fig. 2). The baseline characteristics of the studied population are presented in Table 1: no significant differences were found between the two groups. Of 27 patients in the protocol group, 1 (3.7%) died in the ICU, 24 (88.9%) were successfully weaned from tracheostomy (16 at first attempt) and transferred to the ward, other ICU or rehabilitation, and 2 (7.4%) were transferred still on the ventilator (Table 2). Of 38 patients in the control group, 2 (5.3%) died in the ICU, 22 (58%) were successfully weaned from tracheostomy (12 at first attempt) and transferred to the ward, other ICU or rehabilitation, and 14 were transferred still on the ventilator (36.8%).

**Table 1:** Characteristics of the study population

	Protocol group	Control group	*p* value
Number of patients	27	38	
Gender males (%)	22 (81.5%)	25 (65.8%)	0.27
Age	61.3 (13.6)	61.7 (15.2)	0.91
*Pathologies at admission*			
Neurological (%)	14 (51.9%)	23 (60.5%)	
Cerebral hemorrhage	5	8	0.66
Cardiac arrest	3	5	
Head trauma	3	3	
CNS infections	1	2	
Ischemic stroke	1	2	
Others	1	3	
Non-neurological (%)	13 (48.1%)	15 (39.5%)	
Urgent surgery	6	2	
Respiratory failure	2	6	
Septic shock	2	2	
Polytrauma	1	4	
Others	2	1	
SAPS II, mean (SD)	47.7 (15.3)	49.8 (14.8)	0.66

*CNS* central nervous system

**Table 2:** Outcome of the study population

	Protocol group (*n* = 27)	Control group (*n* = 38)	*p* value
Discharged to the ward (all weaned)	21 (77%)	17(45%)	0.031*
*Discharged to other ICU*			
Weaned	2 (7%)	2 (5%)	
Not weaned	1 (4%)	0 (0%)	
*Discharged to rehabilitation*			
Weaned	1 (4%)	3 (8%)	
Not weaned	1 (4%)	14 (37%)	
Patients dead in the ICU	1 (4%)	2 (5%)	
ICU length of stay (days), mean (SD)	19.0 (7.5)	21.1 (9.2)	0.35

*ICU* intensive care unit

*p < 0.05 compared to protocol group

Concerning the feasibility results, no patients were excluded after randomization. There was no crossover between the two study arms. We did not have any missing data, and no severe adverse event related to the study protocol application was recorded by the staff.

Start of weaning timing and M-BWAP scores, TI, PSV, PEEP and PaO_2_/FiO_2_ at recruitment did not differ between groups. Control group patients had a significantly higher risk of being discharged from the ICU while still mechanically ventilated compared to protocol group (2 of 26 ICU survivors versus 14 of 26; relative risk: 1.5, IC 95% 1.14–2.01). No differences were found concerning the ICU-LOS (Table 3).

**Table 3:** Measured parameters of the mechanical ventilation and weaning process

	Protocol group (*n* = 27)	Control group (*n* = 38)	*p* value
Timing of tracheostomy (days after ICU admission), median (IQR)	7 (4–8.5)	5 (4-7)	0.23
Weaning start (days after tracheostomy), mean (SD)	2.4 (2.4)	3.8 (3.5)	0.416
M-BWAP at recruitment, mean (SD)	14.7 (2.0)	14.5 (2.4)	0.71
Tobin Index at recruitment, mean (SD)	53 (26)	67 (38)	0.33
PSV at recruitment (cmH_2_O), mean (SD)	10.2 (2.6)	11.2 (2.6)	0.16
PEEP at recruitment (cmH_2_O), mean (SD)	9.0 (2.0)	9.3 (2.1)	0.59
PaO_2_/FiO_2_ at recruitment, mean (SD)	277 (67)	290 (75)	0.46

*M-BWAP* modified Burns Weaning Assessment Program, *PSV* pressure support ventilation, *PEEP* positive end-expiratory pressure

*p < 0.05 compared to protocol group

**Fig. 3:**
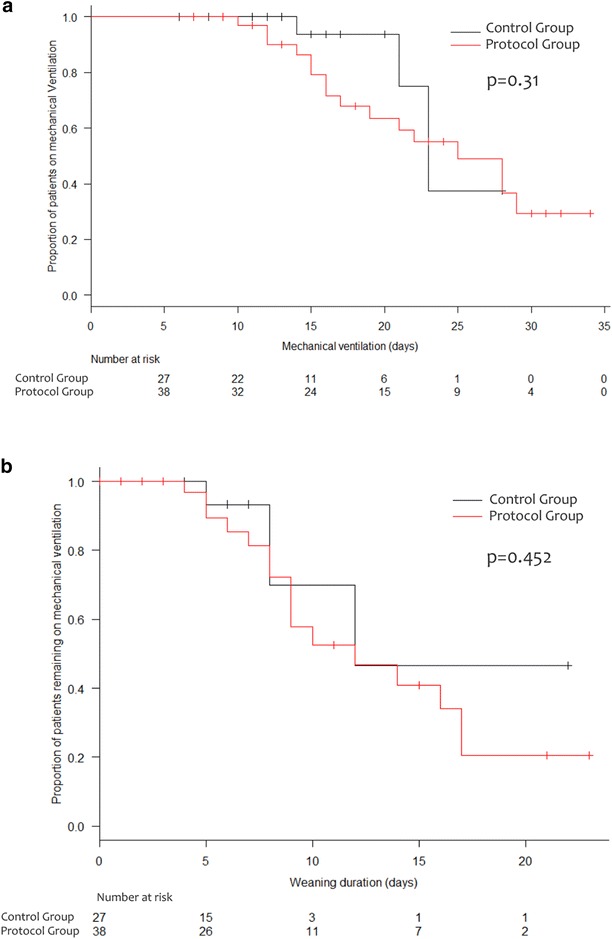
Kaplan–Meier and log-rank test for MV duration (**a**) and weaning time (**b**) in the two study groups. Patients discharged from the ICU still ventilated or dead were censored

The Kaplan–Meier curves censoring patients who died in the ICU or who were discharged still ventilated [16 (42.1%) patients in the control group and 3 (11.1%) patients in the protocol group] showed no difference between the two study groups regarding weaning time (*p* = 0.45) and duration of MV (*p* = 0.31) (Fig. 3). In the secondary analysis, where the patients still ventilated at ICU discharge were considered as weaned, the weaning time and duration of MV were significantly shorter and successful weaning rate was significantly higher in the protocol group compared to the control group (Additional file 1: eTable 2).

Based on our results and assuming a 30% reduction of the weaning time for the protocol group, a study power of 90% and *α* error of 0.05, 280 patients would be needed for a RCT to establish efficacy.

## Discussion

The present pilot RCT demonstrated that a nurse-led weaning protocol compared to weaning based on physician’s clinical judgment could reduce the weaning time and the total duration of MV, and increased successful weaning rate in tracheostomized critically ill patients. Moreover, a multicenter RCT would be feasible and would require 280 patients to prove efficacy.

Feasibility was established based on the ability of the ICU team to recruit a sufficient number of patients and collect data, to have a low crossover rate between the two study arms, and good safety of protocol application. We had no crossovers nor any missing data, despite the complexity of the M-BWAP, probably due to the strong motivation of nurses to complete the data collection. There were no severe adverse events indicating that the protocol was safely implemented.

We enrolled 65 patients in 1 year, which is a good result considering the estimated 280 patients needed for a large RCT to definitely prove efficacy. Vaschetto et al. [20] showed similar rate in the only other RCT performed in tracheostomized critically ill patients by enrolling 168 patients in a 3-year period in a single ICU in Italy. An RCT of adequate power could be completed in 18 months with participation of six ICUs with comparable case mix and tracheostomy policy, each enrolling 50 tracheostomized patients per year. These results support the feasibility and acceptability of conducting a larger randomized controlled trial including outcome missing in the present study: the decannulation rate, the ventilator-free days, along with 28- and 90-day mortality of the patient discharged from the ICU. Although no statistical differences were found in the ICU-LOS, 2-day difference is what resulted from the data concerning the weaning period.

Considering the secondary outcome, we could notice a reduced MV duration and weaning time in the protocol group compared to the control group, although non-statistically significant. In fact, our conclusion is based on the Kaplan–Meier with log-rank test, censoring all patients died in ICU or discharged still ventilated from the ICU. This approach leads to a reduced number of patients analyzed, under powering our trial. In the sensitivity analysis, considering patients discharged still ventilated as weaned, giving a greater advantage to the control group where those patients are more represented (16 patients vs. 2 patients in the protocol group), the differences in MV duration and weaning time became highly significant.

Vaschetto et al. [19] conducted their RCT in a population of critically ill neurological and neurosurgical patients and found no difference in reconnection rate to MV comparing daily screening of readiness for SBT based on physiologic and clinical variables followed by SBT with direct disconnection from the ventilator based on physician’s judgment, the same control arm we adopted in our trial. The study was interrupted after inclusion of 168 patients because reconnection rates were much higher than anticipated (29% instead of 16%), and the authors estimated that a further trial should overall enroll 790 patients. This is a substantially higher than our estimation of 280 patients. Sample size calculation is greatly influenced by the predicted effect size of the treatment under study compared to the control treatment on the outcome [37], suggesting that the proposed nurse-led weaning protocol might be superior to daily screening of clinically relevant variables. The M-BWAP is a comprehensive clinical weaning checklist and scoring instrument; moreover, once the M-BWAP was judged as adequate, the nurse took charge of the entire weaning process with strict patient follow-up at the bedside allowing fine-tuning and timely modification of pressure support ventilation and PEEP in accordance with the protocol steps.

Weaning process was started a mean of 1.6 days earlier in the protocol group than in the control group, although this difference was not statistically significant. We speculated that stricter application of the M-BWAP score in the protocol group may have played a role, because the weaning process started immediately after a predetermined score was reached, whereas in control group physicians started the weaning by clinical judgments whenever it was considered appropriate.

The nurses judged the protocol as being easy to implement. We did not perform a formal survey to explore this aspect, but nurse performance was good as they completely adhered to the protocol without any loss of patient data nor crossover.

A note has to be made regarding the early tracheostomy approach we have adopted in our patients. Although the time to tracheostomy was not an outcome in our study, we should notice the short period between the start of MV and tracheostomy with a median (IQR) of 7 (4–8.5) days in the protocol group and 5 (4–7) days in the control group. Even if the literature is not conclusively in favor to an early approach to the tracheostomy, we have traditionally adopted this approach as standard of care mainly because the unit admits large numbers of neurocritical care patients (up to one-fourth in the control group), in whom incidence of VAP is high [38], and early tracheostomy may provide some benefits [39]. Over the years, this approach has been extended to non-neurological patients since early tracheostomy has been associated with lower incidence of ventilator-associated pneumonia and a significant reduction of MV duration [40]. A recent Cochrane’s review with meta-analysis [41] confirmed these results, although not conclusively.

Moreover, we based the clinical decision to perform a tracheostomy on the prevision of prolonged MV or a prevision of difficult and prolonged weaning. Although our decision was not based on a precise protocol, the attitude of the ICU physician coordinator regarding the tracheostomy has been based on the literature. In particular, pneumonia, acute respiratory distress syndrome (ARDS), neuromuscular disease, head trauma and intracerebral hemorrhage patients have a longer mechanical ventilation [42]. Age and obesity are the main demographic characteristics related to prolonged MV, and history of COPD, PaO_2_/FiO_2_ at intubation, lung injury severity score (LIS) [43] and SOFA [28] at admission, are the main variables related to prolonged MV. COPD [30, 43], age [30, 43], SOFA at admission, the presence of pneumonia and sepsis, ARDS and high level of PEEP during the first failed SBT [30], are the variables associated with a prolonged and difficult weaning.

Last consideration should be made on the applicability of a weaning protocol by nurses; considered all together, the results of our study demonstrated that a well-designed and shared protocol could be entrust to the nurses, especially in countries where the respiratory therapists are not available.

### Limitation of the study

Firstly, the study was not blinded to treatment allocation. Thus, nurses might have been motivated to wean patients assigned to the protocol group; however, this effect might be counterbalanced by similar motivations in physicians for the control group. At the same time, physicians and nurses were aware that they were being observed and might have been more diligent in managing their patients. In fact this ‘trial effect’ is well described as the phenomenon of improved health outcome in patients treated with standard of care on trial compared to those receiving standard of care outside of a clinical trial setting [38]. A cluster RCT with randomization of participating ICUs to protocol or control would be an option for future RCT, as this would make blinding useless. Moreover, cluster RCTs are suggested to be the best choice whenever the intervention might be a treatment that requires health professionals to change their behavior to impact patient outcomes. However, a cluster RCT would require a larger sample size than that of a non-cluster trial. Hence, researchers should balance the advantage of blinding and homogeneous behavior with the shortcoming of increased resource demand.

Secondly, the patients’ observation period did not extend outside the ICU, with 16 patients (2 in the protocol and 14 in the control group) still on the ventilator at the time of ICU discharge. This is *per sé* a proof of efficacy because patients in the protocol group were more frequently weaned than patients in the control group despite comparable weaning start timing and duration of ICU stay. Further, we considered these patients as weaned in the sensitivity analysis, thus favoring the control group.

Future studies should consider extending the observation period until effective withdrawing from MV. Effective patient decannulation and three-month mortality rate would also be worth considering in a future RCT.

Moreover, we enrolled a mixed population of neurological and non-neurological ICU patients. Indication for tracheostomy and causes of failed weaning may differ in these two populations. However, demonstration of protocol efficacy in these diverse populations would strengthen generalizability of results.

A specific protocol to perform early tracheostomy was not adopted, and the decision was based on a consensus between ICU physicians as previously stated, mainly based on clinical prediction of prolonged MV or difficult or prolonged weaning [26], although the actual condition of the patients did not met the criteria (frequently leading to an early tracheostomy). Moreover, the M-BWAP is a non-validated score. The points 20–21–23–25 of the original BWAP were not considered, since these variables required patient’s collaboration, which is often lacking in the acute stage of critical illness. The choice of M-BWAP > 15 and BWAP 10–15 with a Tobin Index < 100 was arbitrary and never compared with the performance of the BWAP. We would like to propose a simple tool to be applied by nurses. To be more conservative, before starting the weaning process, we decided that at least 70% of the items should be met before starting the weaning process (M-BWAP > 16).

A note should be made on the sample size calculation for a future RCT, since the number resulted (280 patients) is based on the result of the present pilot study with the above-mentioned limitations, in particular the application of an early tracheostomy and an absence of post-ICU data.

As a last limitation, although a computer-based simple allocation was used, the number of patients per arms resulted unbalanced.

## Conclusions

With this pilot study we demonstrated that a protocolled approach to weaning tracheostomized patients could be able to reduce the weaning time and the total duration of MV, and increased successful weaning rate in tracheostomized critically ill patients. Moreover, a larger multicenter RCT comparing a nurse-led weaning protocol with a weaning based on physician’s clinical judgment would be feasible and would require 280 patients to definitely prove efficacy.

## Additional file

**Additional file 1. Table S1:** Modified Burns Weaning Assessment Program (M-BWAP) checklist. **Table S2**: Sensitivity analysis for weaning time, duration of MV and successful weaning, considering patients still ventilated at ICU discharge as weaned. **Figure S1**: Schematic presentation of the of the weaning process in the orotracheal intubated patients and in the control group.

## Abbreviations

BWAP: Burns Wean Assessment Program score
ICU: intensive care unit
ICU-LOS: intensive care unit length of stay
M-BWAP: modified Burns Wean Assessment Program score
MV: mechanical ventilation
PEEP: positive end-expiratory pressure
PSV: pressure support ventilation
RCT: randomized controlled trial
SAT: sedation awakening trial
SBT: spontaneous breathing trial
TI: Tobin Index
TV: tidal volume
VAP: ventilator-associated pneumonia
