# Supplementary material for: Comparison between a nurse-led weaning protocol and weaning based on physician’s clinical judgment in tracheostomized critically ill patients: a pilot randomized controlled clinical trial
Source: Ann Intensive Care. 2018 Jan 22;8:11. doi: 10.1186/s13613-018-0354-1 (PMC5778092; doi:10.1186/s13613-018-0354-1)
Supplement: Supplementary file 1 — Additional file 1. Table S1: Modified Burns Weaning Assessment Program (M-BWAP) checklist. Table S2: Sensitivity analysis for weaning time, duration of MV and successful weaning, considering patients still ventilated at ICU discharge as weaned. Figure S1: Schematic presentation of the of the weaning process in the orotracheal intubated patients and in the control group. [file 13613_2018_354_MOESM1_ESM.doc]

**Comparison between a nurse-led weaning protocol and weaning based on physician’s clinical judgment in tracheostomized critically ill patients:**

**a pilot randomized controlled clinical trial.**

**Additional file note**

The Modified-Burns Wean Assessment Program (M-BWAP) score is a BWAP score without four items: negative inspiratory pressure (item 20), positive expiratory pressure (21), vital capacity (23), and PaCO_2_ with minute ventilation <10 L/min (item 25). Since each item was worth one point, the maximum score decreased from 26 to 22. Moreover, the item “PaO_2_ > 60 mmHg on FiO_2_ < 40 %” was substituted by the ratio between the arterial partial pressure of oxygen and the inspired fraction of oxygen (PaO_2_/FiO_2_) > 200 (Table S1).

The application of a modified BWAP score was also proposed by Jiang and colleagues (2014), however, we considered their model as being too complicated to be applied routinely. Moreover, it was applied to intubated patients without tracheostomy (Jiang et al., 2014). The M-BWAP score we used was meant to be easily applied, thus the items related to measurement of lung volumes and exerted pressure were not used; furthermore, as this score was also applied in neurological patients, most of them could not perform these evaluations.

REFERENCES

1. Burns SM, Fisher C, Earven Tribble SS, Lewis R, Merrel P, Conaway MR, Bleck TP. Multifactor clinical score and outcome of mechanical ventilation weaning trials: Burns Wean Assessment Program. Am J Crit Care. 2010 Sep;19(5):431-9. doi: 10.4037/ajcc2010273.
2. Jiang JR, Yen SY, Chien JY, Liu HC, Wu YL, Chen CH (2014) Predicting weaning and extubation outcomes in long-term mechanically ventilated patients using the modified Burns Wean Assessment Program scores. Respirology 19:576-82.

| **Modified BURNS' WEAN ASSESSMENT PROGRAM (M-BWAP)** | | |
| --- | --- | --- |
| **YES** | **NO or not assessed** | **GENERAL ASSESSMENT** |
| □ | □ | 1. Hemodynamically stable? (Pulse rate, cardiac output) |
| □ | □ | 1. Free from factors that increase or decrease metabolic rate? (seizures, temperature, sepsis, bacteremia, hypo/hyper thyroid) |
| □ | □ | 1. Hematocrit > 25% (or baseline)? |
| □ | □ | 1. Systemically hydrated (weight at or near baseline, balanced intake and output)? |
| □ | □ | 1. Nourished? (albumin > 2.5, parenteral/enteral feedings maximized) If albumin is low and anasarca is present, score for hydration should be "no." |
| □ | □ | 1. Electrolytes within normal limits? (including Ca^++^, Mg^+^, PO_4_). |
| □ | □ | 1. Pain controlled? (subjective determination) |
| □ | □ | 1. Adequate sleep/rest? (subjective determination) |
| □ | □ | 1. Appropriate level of anxiety and nervousness? (subjective determination) |
| □ | □ | 1. Absence of bowel problems? (diarrhea, constipation, ileus) |
| □ | □ | 1. Improved general body strength/endurance? (i.e., out of bed in chair, progressive activity program) |
| □ | □ | 1. Chest x-ray improving or returned to baseline? |
|  |  | **RESPIRATORY ASSESSMENT** |
| □ | □ | 1. Eupneic respiratory rate and pattern (RR <25, without dyspnea, absence of accessory muscle use). |
| □ | □ | 1. Absence of adventitious breath sounds? (rhonchi, rales, wheezing) |
| □ | □ | 1. Secretions thin and minimal? |
| □ | □ | 1. Absence of neuromuscular disease/deformity? |
| □ | □ | 1. Absence of abdominal distention/obesity/ascites? |
| □ | □ | 1. Oral endo tracheal tube ≥ 7.5 or tracheostomy ≥ 6.0 (I.D.) |
| □ | □ | 1. Cough and swallow reflexes adequate? |
| □ | □ | 1. Spontaneous tidal volume > 5 ml kg^-1^? |
| □ | □ | 1. pH 7.30-7.45 |
| □ | □ | 1. PaO_2_/FiO_2_ >200 |

**Table S1.** Modified Burns Weaning Assessment Program (M-BWAP) checklist. Compared to the original version, the M-BWAP does not include the items: 20, 21, 23 and 25. The original item numbered 26, and now 22 was: PaO_2_ >60 mmHg with fraction of inspired oxygen <40. Each item is worth 1 point. The weaning process started with a M-BWAP score >15 or between 10 and 15 with a Tobin Index <100.

|  | **Protocol Group**  **(n=27)** | **Control Group**  **(n=38)** | ***p-value*** |
| --- | --- | --- | --- |
| **Weaning time (days), mean (SD)** | 5.4 (4.4) | 8.3 (5.7) | *0.029** |
| **Mechanical ventilation (days), mean (SD)** | 14.4 (5.3) | 18.1 (7.9) | *0.039 ** |
| **Successful weaning, number (%)** | 24 (88.9%) | 22 (57.9%) | *0.015 ** |

**Table S2:** Sensitivity analysis for weaning time, duration of MV and successful weaning, considering patients still ventilated at ICU discharge as weaned. *: p<0.05 compared to protocol group.


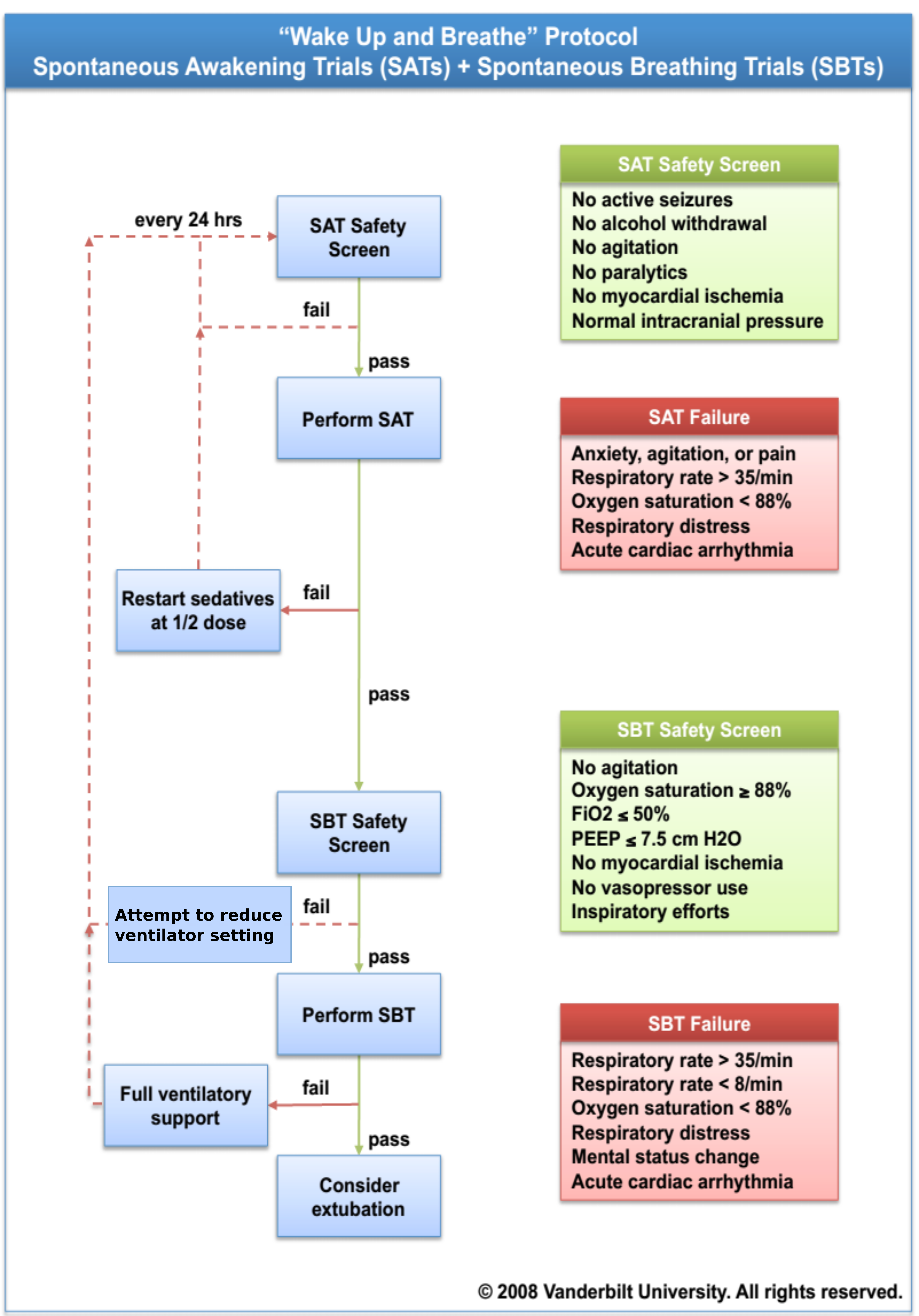


**Figure S1.** Schematic presentation of the of the weaning process in the orotracheal intubated patients and in the control group. Modified from [www.icudelirium.org/docs/WakeUpAndBreathe.pdf](http://www.icudelirium.org/docs/WakeUpAndBreathe.pdf) (Vanderbilt University).
